# Supplementary material for: Smells like γ-synchrony: Insula-prefrontal communication depends on γ-synchrony and supports modality-specific changes in behavioral strategies
Source: bioRxiv. 2026 Aug 12:2026.08.06.743126. Preprint. [Version 1] doi: 10.64898/2026.08.06.743126 (PMC13640584; doi:10.64898/2026.08.06.743126)
Supplement: Supplementary file 1 [file NIHPP2026.08.06.743126v1-supplement-1.pdf]

bioRxiv preprint doi: https://doi.org/10.64898/2026.08.06.743126; this version posted August 12, 2026. The copyright holder for this preprint (which was not certified by peer review) is the author/funder, who has granted bioRxiv a license to display the preprint in perpetuity. It is made available under a CC-BY 4.0 International license.

Supplementary Figures

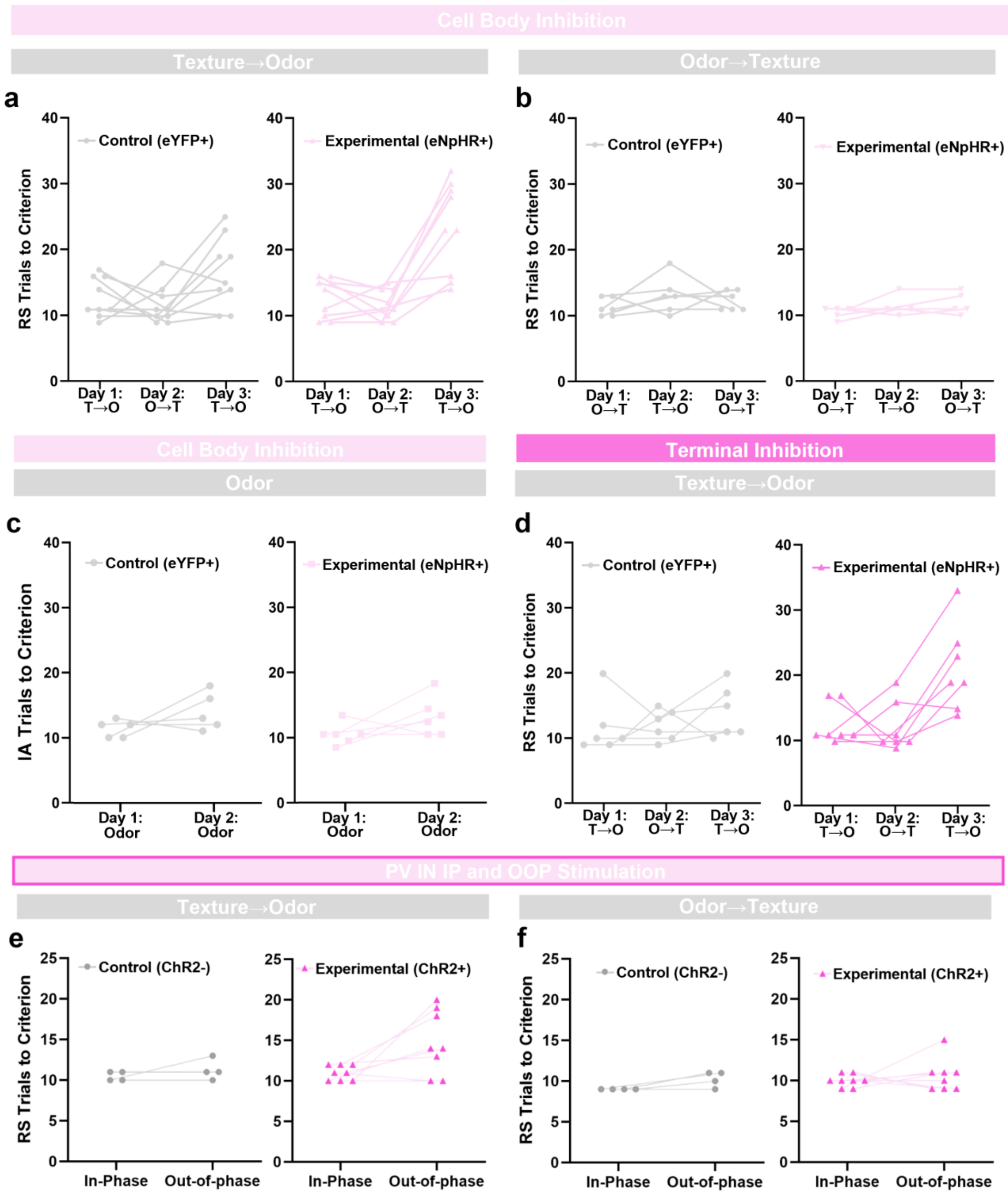

Supplementary Figure 1: Behavior of individual mice during optogenetic manipulations. a, Behavior of mice receiving mIC to mPFC cell body inhibition while shifting from texture → odor on Day 3. **b,** Behavior of mice receiving mIC to mPFC cell body inhibition while shifting from odor → texture on Day 3. **c,** Behavior of mice receiving cell

bioRxiv preprint doi: https://doi.org/10.64898/2026.08.06.743126; this version posted August 12, 2026. The copyright holder for this preprint (which was not certified by peer review) is the author/funder, who has granted bioRxiv a license to display the preprint in perpetuity. It is made available under a CC-BY 4.0 International license.

body inhibition when learning odor-based IA on Day 2. **d,** Behavior of mice receiving mIC to mPFC terminal inhibition while shifting from texture → odor. **e,** Behavior of mice when shifting from texture → odor during IP and OOP excitation of PV INs in mIC and mPFC. **f,** Behavior of mice when shifting from odor → texture during IP and OOP excitation of PV INs in mIC and mPFC.

bioRxiv preprint doi: https://doi.org/10.64898/2026.08.06.743126; this version posted August 12, 2026. The copyright holder for this preprint (which was not certified by peer review) is the author/funder, who has granted bioRxiv a license to display the preprint in perpetuity. It is made available under a CC-BY 4.0 International license.

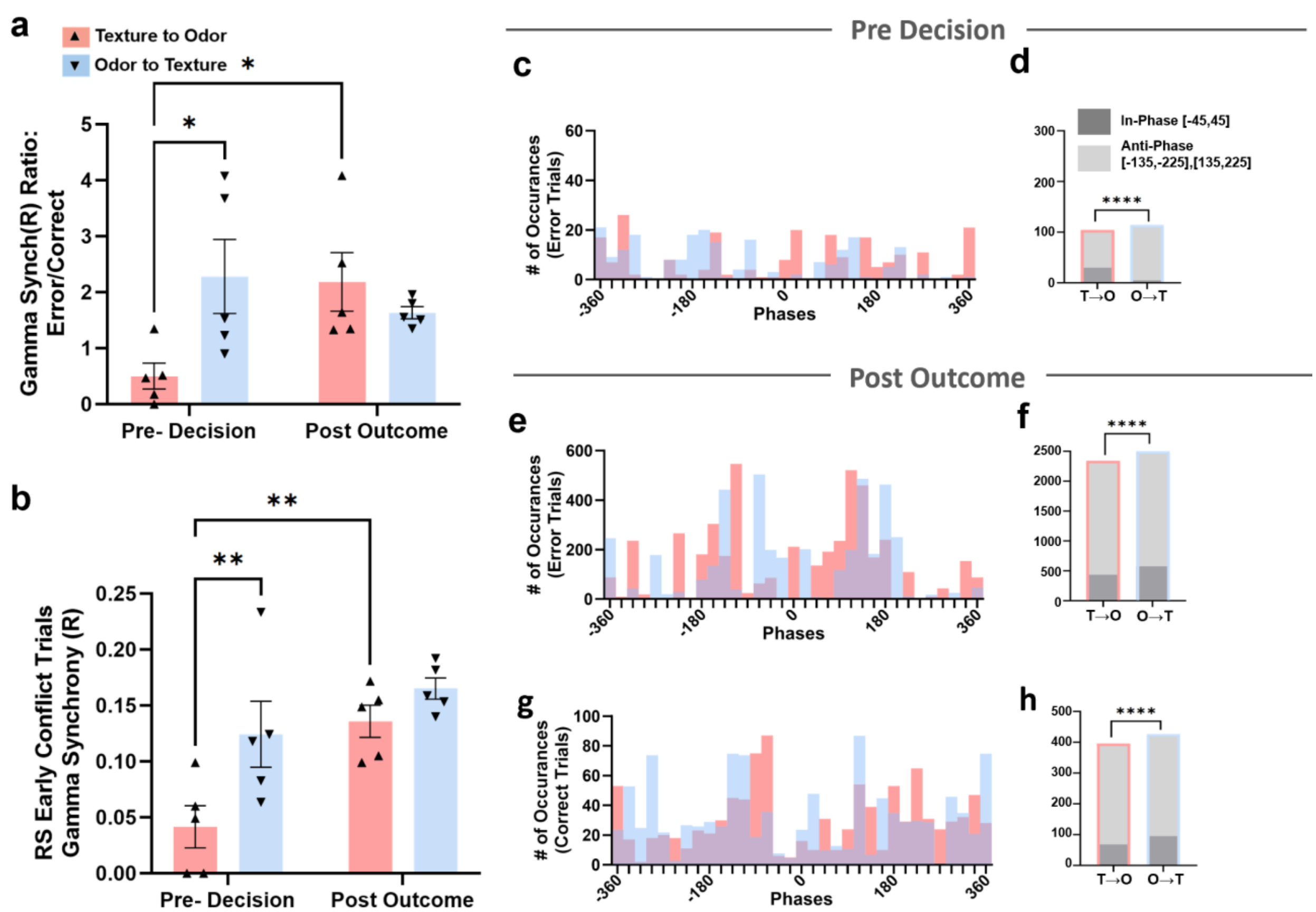

Supplementary Figure 2: mIC-mPFC PV IN gamma synchrony changes depending on whether mice shift to odor or texture-based strategies. a, The ratio of gamma synchrony on error vs. correct trials during the pre-decision or post-outcome period for different types of rule shifts. **b,** Gamma synchrony during the pre-decision or post-outcome period of early conflict trials (i.e., conflict trials within the first 5 trials) during different types of rule shifts. **c,** Distribution of mIC-mPFC phase differences (locations of the peak cross-correlation value) during the pre-decision period on error trials (calculated for phase shifts from -360 to 360 degrees). **d,** Comparison of the fraction of in-phase (~0 degree) vs. anti-phase (~180 or -180 degree) phase differences during the pre-decision period for texture to odor vs. odor to texture shifts. **e-h,** Comparison of phase differences during post-outcome periods. **e,** Similar to (c) but for the post-outcome period of error trials. **f,** Similar to (d) but for the post-outcome period of error trials. **g,** Similar to (c) but for the post-outcome period of correct trials. **h,** Similar to (d) but for the post-outcome period of correct trials. * $p < 0.05$. ** $p<0.01$. **** $p < 0.0001$.

bioRxiv preprint doi: https://doi.org/10.64898/2026.08.06.743126; this version posted August 12, 2026. The copyright holder for this preprint (which was not certified by peer review) is the author/funder, who has granted bioRxiv a license to display the preprint in perpetuity. It is made available under a CC-BY 4.0 International license.

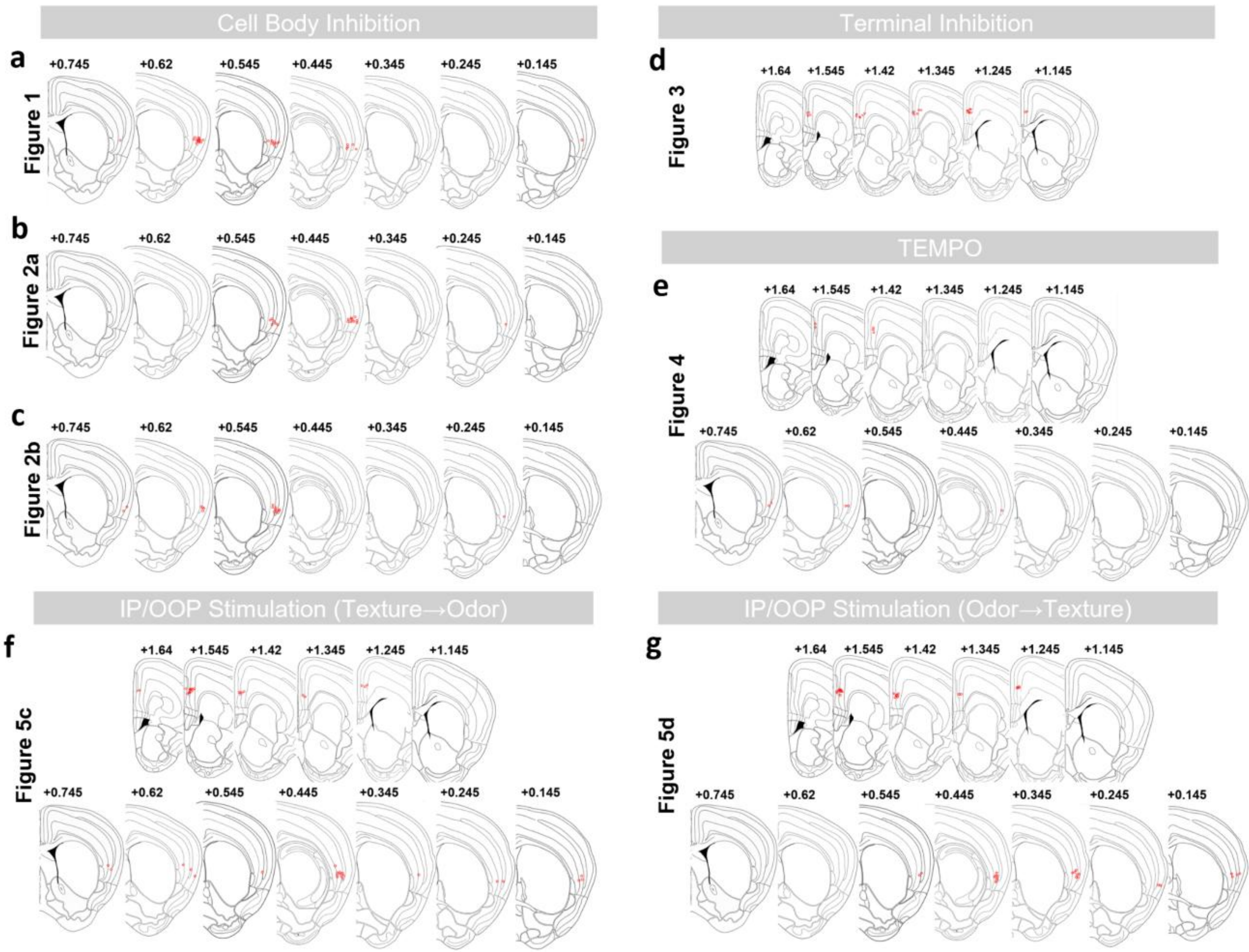

Supplemental Figure 3: Locations of fiber optic implants. **a-c,** Implant locations within the mIC for cell body inhibition experiments shown in Figures 1 and 2. **d,** Implant locations within the mPFC for mIC to mPFC terminal inhibition experiment shown in Figure 3. **e,** Implant locations within the mIC and mPFC for TEMPO recordings shown in Figure 4. **f-g,** Implant locations within the mIC and mPFC for optogenetic stimulation experiments shown in Figure 5.

bioRxiv preprint doi: https://doi.org/10.64898/2026.08.06.743126; this version posted August 12, 2026. The copyright holder for this preprint (which was not certified by peer review) is the author/funder, who has granted bioRxiv a license to display the preprint in perpetuity. It is made available under a CC-BY 4.0 International license.

Supplemental Table 1: Summary of Statistics for Supplemental Figure 2

Panel	Time Period	Statistical test			Multiple comparison	
**Supplemental Figure 2a**	**Pre-Decsion and Post Outcome**	**2-Way ANOVA**			**Comparison**	**P-value**
		**Source of Variation**	**P value**	**F (DFn, DFd)**	Pre- Decision	
		Interaction	0.0175	F (1, 16) = 7.015	Texture to Odor Shift vs. Odor to Texture Shift	0.0113
		Phase Shift	0.255	F (1, 16) = 1.394		
		Behavior Shift	0.1816	F (1, 16) = 1.950	Post Outcome	
					Texture to Odor Shift vs. Odor to Texture Shift	0.3891
					Texture to Odor Shift	
					Pre- Decision vs. Post Outcome	0.0155
					Odor to Texture Shift	
					Pre- Decision vs. Post Outcome	0.3147
**Supplemental Figure 2b**	**Pre-Decsion and Post Outcome**	**2-Way ANOVA**			**Comparison**	**P-value**
		**Source of Variation**	**P value**	**F (DFn, DFd)**	Pre- Decision	
		Interaction	0.1897	F (1, 16) = 1.876	Texture to Odor Shift vs. Odor to Texture Shift	0.0085
		Phase Shift	0.0032	F (1, 16) = 11.99		
		Behavior Shift	0.0111	F (1, 16) = 8.246	Post Outcome	
					Texture to Odor Shift vs. Odor to Texture Shift	0.304
					Texture to Odor Shift	
					Pre- Decision vs. Post Outcome	0.0035
					Odor to Texture Shift	
					Pre- Decision vs. Post Outcome	0.1584
**Figure 2d**	Pre-Decsion Correct Trials	**Chi-square**			NA	NA
		**Comparison**	**Sides**	**P value**		
		In-Phase [-45, 45] vs Anti-Phase [-135,-225] vs Anti-Phase [135,225]	Two	<0.0001		
**Figure 2e**	Pre-Decsion Correct Trials	**Chi-square**			NA	NA
		**Comparison**	**Sides**	**P value**		
		In-Phase [-45, 45] vs Anti-Phase [-135,-225] vs Anti-Phase [135,225]	Two	<0.0001		
**Figure 2f**	Pre-Decsion Correct Trials	**Chi-square**			NA	NA
		**Comparison**	**Sides**	**P value**		
		In-Phase [-45, 45] vs Anti-Phase [-135,-225] vs Anti-Phase [135,225]	Two	<0.0001		
